# Pharmacist Outlooks on Prescribing Hormonal Contraception Following Statewide Scope of Practice Expansion

**DOI:** 10.3390/pharmacy7030096

**Published:** 2019-07-18

**Authors:** Sally Rafie, Emily Richards, Samantha Rafie, Sharon Cohen Landau, Tracey A. Wilkinson

**Affiliations:** 1 Department of Pharmacy, UC San Diego Health, San Diego, CA 92103, USA; 2 Birth Control Pharmacist, San Diego, CA 92122, USA; 3 Safeway Pharmacy, Santa Rosa, CA 95403, USA; 4 Mental Health Service, VA San Diego Healthcare System, San Diego, CA 92161, USA; 5 Children’s Health Services Research, Department of Pediatrics, Indiana University School of Medicine, Indianapolis, IN 46202, USA

**Keywords:** qualitative research, pharmacists, contraception, pharmacy access, pharmacies

## Abstract

In an effort to increase access to contraception, the pharmacist scope of practice is being expanded to allow prescribing. While this is being accomplished in the United States by a variety of models, legislation that allows pharmacists to prescribe hormonal contraception under a statewide protocol is the most common. This study was designed to explore the outlooks of pharmacists regarding prescribing contraception in the period following the first state legislation and prior to statewide protocol development and availability. A qualitative study of community pharmacists in California using structured phone interviews explored their opinions regarding access to contraception in pharmacies and outlooks regarding prescribing. Data were analyzed using an inductive approach to identify themes. Among the thirty participants, the majority worked in a chain pharmacy. Themes were identified in five overarching domains: Pharmacist barriers, system barriers, patient issues, safety concerns, and pharmacist role. Most were unfamiliar with the new law, yet were interested in expanding access for patient benefit despite foreseeing challenges with implementing the service in community pharmacies. Barriers will need to be addressed and requisite training disseminated widely to facilitate successful implementation and thus improve access on a broad scale. Further research following protocol implementation is needed to understand service implementation, as well as patient utilization and satisfaction.

## 1. Introduction

There are many challenges to birth control access in the United States (US), including costs of clinic visits, difficulties getting an appointment, required annual checkups, the time required for clinic visits, restrictions on the number of packs of prescription contraceptives dispensed, and the limited period in which prescriptions can be refilled [1]. One strategy is to create additional access points at community pharmacies by expanding the pharmacist scope of practice at the state level to include prescribing of contraception.

In a survey of US women, 67% indicated that they would personally benefit from not having to pay for a doctor or clinic visit if they could access hormonal contraception directly at the pharmacy [2]. In addition, 85% indicated that they would benefit from the convenient locations and hours of community pharmacies because of the saved time and lack of a required appointment [2]. Since community pharmacists are uniquely accessible and commonly used as first-line medical advisors, they are also in a position to provide education and clinical services for contraception, including education and referrals for long-acting reversible contraceptives (LARC) [3].

While over-the-counter contraception has been discussed for nearly 20 years and exists in other countries, all hormonal contraceptives with the sole exception of levonorgestrel emergency contraceptive pills remain prescription-only in the United States. While the prescription-only status remains unchanged, states have begun expanding their pharmacist scope of practice to include prescribing of contraception under statewide collaborative practice agreement or protocol or dispensing under statewide standing order [4]. States can also allow for individual collaborative practice agreements.

The first state to specifically expand this authority was California [5]. California’s law became effective in 2014 and permitted its pharmacists to opt-in to prescribe and dispense contraceptives directly to patients of all ages following a statewide protocol to be subsequently developed by the California Board of Pharmacy [5]. There were no resources for implementation and the law did not address payment for the pharmacist’s service. The California Board of Pharmacy began protocol development in 2014 and in 2016, the statewide protocol became available for pharmacist use following a minimum of one hour of training, which is available from all California schools of pharmacy and multiple continuing education providers. The protocol requires use of a patient self-screening questionnaire to determine eligibility for the various self-administered hormonal contraceptive methods—pill, transdermal patch, vaginal ring, and depot medroxyprogesterone acetate (DMPA) injection. In the first year after protocol availability, 5%–11% of pharmacies were providing the service [6,7].

While there are surveys of pharmacist interest and intentions to provide this service along with their motivations and barriers, there are no qualitative data on pharmacists’ outlooks about participating in prescribing hormonal contraception following legislative expansion in their scope of practice that allows it [8,9,10,11]. The objective of this study was to explore pharmacists’ outlooks regarding the forthcoming ability to prescribe hormonal contraception in the period after the law became effective and prior to statewide protocol availability. Since the vast majority of pharmacies are not offering this service, this study will help inform ongoing implementation efforts. Similar protocols have since been implemented in several other states with more following each year [12]. There is generally a noteworthy lag time (i.e., 2.5 years in California) from legislation passing to regulation and protocol development and approval. For this reason, the findings of this study are relevant beyond California and can be incorporated in policy design and implementation efforts in other states.

## 2. Materials and Methods 

This is a qualitative interview study of community pharmacists in California. A comprehensive list of all pharmacies was generated on 29 March 2014 using the California Board of Pharmacy public website and filtered to retain only 6353 community pharmacies with active licenses. A random number generator determined the sequence in which the pharmacies were called to recruit pharmacists to participate in an interview. Recruitment efforts were terminated when 30 participants were recruited and thematic saturation was achieved. Each participant completed a brief demographic survey before their interview.

The interviews were conducted by phone from July to August 2014 by a single investigator (ER) using a semi-structured interview guide and audio recorded digitally. The interview guide was designed by the investigators, nearly all of whom have been involved with training, service implementation, and prior research in this area. It consisted of 21 open-ended questions to generate discussion on pharmacist prescribing of hormonal and emergency contraception, including sample patient vignettes to prompt additional discussion: (1) Repeat patient coming in for emergency contraception on multiple occasions, (2) patient is late refilling her birth control and has no refills left, (3) patient is filling a prescription for a new medication, topiramate, and you notice she has birth control pills on her profile, (4) an obese woman asking for emergency contraception, and (5) 17 year-old is interested in starting contraception (Appendix A). 

Interviews lasted approximately 20 to 30 min. Participants received a $20 electronic gift card incentive upon completion of the interview.

Interview responses were linked to demographic information using participant numbers, such that no personal identifiers were linked to responses at the time of data analysis. Each interview was transcribed and proofread for accuracy. The responses were coded inductively using a set of preliminary themes arising from interviews, which were expanded upon as additional themes emerged during analyses. All qualitative data were analyzed using the independent two-coder method and any discrepancies were discussed and reconciled. Themes were determined and exemplary quotations chosen. The University of California San Diego Human Research Protections Program institutional review board approved the study materials and procedures.

## 3. Results

### 3.1. Participants

A total of 146 community pharmacies were contacted during recruitment, 35 (24%) pharmacists agreed to participate, and 30 pharmacists completed interviews before thematic saturation was achieved. The most common reason given when declining to participate was the potential participant was too busy. There was equal representation among genders and pharmacist roles. Participants’ demographic characteristics are presented in Table 1.

A majority (66%) of pharmacists were not aware of the recent state legislation expanding pharmacist scope to include prescribing hormonal contraception. Participants support pharmacist prescribing of most contraceptive methods (90% for progestin-only pills, 90% for combined-oral contraceptives, and 83% for depot medroxyprogesterone acetate) with adequate training and protocols in place. Nearly two-thirds (63%) of the participants intend to participate once the protocol becomes available. 

Themes were identified in five overarching domains. Themes are described along with representative quotations.

### 3.2. Pharmacist Barriers

Pharmacists discussed various barriers that could prevent them from providing hormonal and emergency contraception services to patients. Pharmacist barriers included knowledge gaps, additional training requirements, and personal objections. 

#### 3.2.1. Pharmacist Knowledge Gap

Knowledge gaps regarding contraception was a primary theme. The knowledge gap was primarily described as not knowing how to adjust therapy in response to the vignettes, while the knowledge gap concerning long-acting reversible methods was often due to inexperience.

*“I don’t ever speak about [long-acting reversible contraception] because I am just not as familiar with those as I am with oral contraceptives.”*

*—Pharmacy manager, rural chain pharmacy*

#### 3.2.2. Burden of Additional Training Requirements

Participants noted that a lack of time or resources hindered access to training required in order to prescribe hormonal contraception. In some cases, the pharmacist stated their desire to obtain training, despite adequate time to do so.

*“I would like to be [trained] someday; I just haven’t had the time to do it.”*

*—Staff pharmacist, urban chain pharmacy*

#### 3.2.3. Religious/Personal Objections

Responses in this theme were mostly personal objections that were rooted in personal religious beliefs or the lack of age restrictions.

*“I wouldn’t want to do it for really, really, really young girls—I don’t feel very comfortable that way. I mean for me, it would be like college student, older teen, 16 or 17 and up, something older, where I think the likelihood of them getting taken advantage of is lower than if they were like 13.”*

*—Staff pharmacist, urban chain pharmacy*

At the same time, some participants also revealed an absence of personal objections.

*“I don’t have any reason to deny anybody contraception.”*

*—Pharmacy manager, urban independent pharmacy*

### 3.3. System Barriers

System barriers included challenges in the pharmacy setting as well as the larger healthcare system. Pharmacists expressed concern over current healthcare system practices that either hinder patients from receiving adequate access or deter pharmacists from participating in new policies. 

#### 3.3.1. Challenges with Traditional Prescribers

Participants mentioned that traditional prescriber clinic or office hours—usually Monday through Friday between 8 a.m. and 5 p.m.—make it difficult both for patients to be seen and for pharmacists to contact prescribers for clarifications before dispensing a prescription. Further, participants described situations when they need to contact a prescriber, but a lack of response delayed the pharmacist from providing medications to patients.

*“We will go ahead and fax the doctor. If [the patient] needs it as soon as possible, we also suggest that they give the doctor a call. Because the thing is, when we call, we never actually get to speak to a doctor—we speak to an MA [medical assistant] or a nurse.”*

*—Pharmacy manager, suburban chain pharmacy*

#### 3.3.2. Challenges with Insurance Coverage

Participants discussed how insufficient insurance coverage prevents patients from utilizing services, such as emergency contraception and extended supplies of hormonal contraception.

*“I think the couple challenges are cost—it’s quite expensive—and billing insurance for EC [emergency contraception], as well as other hormonal contraception is an issue. With the ACA [Affordable Care Act], we have seen better co-pays, but I think women should be able to fill them in 3-month supplies so that it is a little bit easier to access and fill the prescription.”*

*—Pharmacy manager, urban chain pharmacy*

Furthermore, participants explained that unless insurance companies provide payment for pharmacist services, there would not be adequate incentive to participate in this new authority. 

#### 3.3.3. Pharmacy Workplace Logistical Barriers

The second most discussed barrier focused on logistical concerns within each pharmacy, mainly a lack of private space to maintain patient confidentiality or time to adequately counsel and complete other essential pharmacy tasks. Other logistical concerns included a lack of equipment to measure blood pressure, insufficient corporate support with pharmacy resources or continuing education, and an inability to store patient records.

*“Sometimes in a community pharmacy setting we don’t have that—you don’t have privacy to begin with and time.”*

*—Staff pharmacist, urban chain pharmacy*

### 3.4. Patient Issues

Patient issues were fundamental in participant discussions. Pharmacists commonly noted concerns related to stigma or patient deficiencies, however, nearly every participant believed the new authority would grant patients greater access and benefit to hormonal contraceptives. 

#### 3.4.1. Patient Self-Management

Pharmacists often expressed that patients were inadequately qualified to self-select and manage contraception in the absence of a healthcare provider guidance (i.e., in an over-the-counter model) due to knowledge gaps, incorrect medication usage, immaturity, or irresponsibility. Participant statements varied from believing patients are often irresponsible, while sexually active to patients not reading medication guides to patients not correctly using contraceptives.

*“I don’t think little girls should be able to just go and buy birth control like that—they are not going to take it every day.”*

*—Staff pharmacist, urban chain pharmacy*

*“The patients are not able to decide whether or not they are a candidate for any [hormonal contraceptives].”*

*—Staff pharmacist, suburban chain pharmacy*

#### 3.4.2. Patient Access and Benefit

Nearly all pharmacists focused on the positive aspects of increased access to contraception. The chief perspective shared by pharmacists was that direct access to contraception in pharmacies outweighs the risks and costs of unintended pregnancy and is beneficial to those patients with less familiarity or access to the healthcare system.

*“I think in the scheme of things, the risk of pregnancy or an unwanted child versus the risk of the hormones and all of the stuff that is already OTC, you can get in trouble with any of it, so if … there would be not stigma, no having to go to the doctor, no Pap smear. I mean I think that would be beneficial for a lot of people.”*

*—Staff pharmacist, suburban chain pharmacy*

#### 3.4.3. Patient Stigma

Some participants considered the shame or embarrassment a patient feels when discussing contraception with their doctor, pharmacist, or parents. One pharmacist emphasized the importance of privacy as a way to lessen patient stigmas.

*“Maybe they are embarrassed to talk to their doctor for fear of their parents finding out or something like that, so if they could do something discreetly in a pharmacy consultation situation, it might be easier for them.”*

*—Staff pharmacist, urban independent pharmacy*

#### 3.4.4. Minors

The issue of access for minors revealed differing opinions to this new prescribing authority. Some pharmacists believed the new authority should allow minors access to contraception without parental involvement, while others believed the opposite.

*“This is an ethics question…She comes to the pharmacy by herself without Mom or Dad…I mean I would dispense it. I wouldn’t have a problem with her age...If her Mom or Dad were present, I would consult them as well as the child or minor in this situation.”*

*—Pharmacy manager, rural chain pharmacy*

*“I would definitely want some rule put in place that the patient has to be 18 and over, unless there is some stipulation that says that we would then by law contact Mom or Dad directly.”*

*—Pharmacy manager, rural chain pharmacy*

One pharmacist believed that adolescents may possibly benefit the most from direct access at the pharmacy.

*“Anyone, but I would say mostly it’s probably the teenagers and in the young 20s. I know that they are resistant to go see the doctor or feel that they can’t trust the doctor or because they are afraid their parents might find out.”*

*—Pharmacy manager, suburban chain pharmacy*

### 3.5. Safety Concerns

Every participant mentioned concern for patient and medication safety at some point during their interview. Along with those concerns was a fear of increased liability for pharmacists providing this service.

#### 3.5.1. Patient Safety Concerns

Safety concerns included difficulty in providing adequate patient counseling, patient neglect of routine health screenings/evaluations, lack of patient history, and inadequate private space in a community setting. 

While this is a broad theme, a common point of discussion centered around how the new authority might allow patients to neglect routine health screenings since they would no longer need to see a traditional provider to obtain hormonal contraception.

*“Why would you go get a pap smear if you can just buy it OTC (over-the-counter)?”*

*—Staff pharmacist, urban chain pharmacy*

Another major safety concern was that hormonal contraceptives can be harmful if incorrectly used or misused, therefore there should be adequate counseling.

*“Birth control can lead to a lot of side effects or a lot of problems in the body.”*

*—Pharmacy manager, suburban chain pharmacy*

#### 3.5.2. Liability Concerns

Participants were often concerned that the new authority could increase their liability, therefore making them hesitant to participate.

*“I have to protect myself and I feel as a pharmacist that I don’t have the full-scope picture of the health of the person to give adequate counseling—that is why I am against it.”*

*—Pharmacy manager, urban clinic pharmacy*

### 3.6. Pharmacist Role

The role of the pharmacist was discussed in these interviews, with approximately half expressing interest in expanding the pharmacist’s role, while the other half believed this new authority was beyond their scope. Participants also shared their current practices and perceived need for pharmacists prescribing of contraception.

#### 3.6.1. Pharmacist Scope

More participants thought pharmacists could take on the new responsibilities than those who did not. Participants shared that the new authority is leading pharmacy practice in a new direction to increase pharmacist roles and responsibilities in providing clinical care.

*“The pharmacist has more of a role and more access in providing care.”*

*—Pharmacy manager, urban chain pharmacy*

Some participants believed prescribing contraception should be left to physicians, usually due to the lack of patient history available in a community pharmacy setting.

*“I can tell her what birth control is, how it works, but I cannot tell her whether or not it is right for her—that is something a prescriber that has her medical history needs to make decisions for.”*

*—Pharmacy manager, suburban chain pharmacy*

#### 3.6.2. Upon Patient Request

Pharmacists explained that they would not proactively intervene or suggest contraception services unless a patient directly asks.

*“It would occur to me to wonder if she had access to a regular form of birth control, but I don’t know if I would really butt in and ask.”*

*—Staff pharmacist, suburban chain pharmacy*

*“I don’t really bring up the long-acting ones just by my initiating it with patients picking up daily oral contraceptive pills.”*

*—Pharmacy manager, suburban chain pharmacy*

#### 3.6.3. Perceived Lack of Need

Pharmacists sometimes felt current models of access to contraception were adequate, and that there was no additional need for pharmacist prescribing. It was noted, for example, that pharmacist prescribing of emergency contraception was not necessary as that is available over-the-counter for people of any age.

*“There is no necessity. There has never been a case where we have had patients who were asking for hormonal contraception without a prescription. The only time that anyone has ever done that is after they have seen the doctor, so there has never been a need for it.”*

*—Pharmacy manager, rural independent pharmacy*

## 4. Discussion

Pharmacists expressed varying outlooks on prescribing hormonal contraception to patients under the forthcoming scope of practice authority. Despite only one-third of the participants being familiar with the law, after being told about it, nearly two-thirds planned to participate. While some pharmacists do not intend to participate, nearly all recognized significant patient benefit with expanded access to contraception. The most common barriers noted were healthcare system barriers and pharmacist knowledge gaps as well as safety concerns; however, all barriers need to be addressed for successful implementation. Safety concerns were discussed by every participant and should receive particular attention during training and other implementation efforts. Many safety concerns brought up are not supported by current evidence and can be addressed with education. In particular, some participants mistakenly believe that pap testing is required annually and is a prerequisite for initiation of hormonal contraception [13,14]. Further, participants reported concerns about side effects with hormonal contraception despite the rate and severity of side effects being lower than many other medications [15].

While it is encouraging that nearly all participants believed this new service would benefit patients through increased contraception access, it is important to note that the major hesitations to provide this service are healthcare system barriers and a pharmacist knowledge gap. While a knowledge gap can be addressed with continuing education for practicing pharmacists and curriculum changes for future pharmacists, system barriers reflect challenges in community pharmacies and the healthcare system as a whole. Overcoming system barriers in the pharmacy may rely on corporate and management support, as well as technology changes. 

Pharmacists prescribing hormonal contraception is a major shift in responsibility and time from dispensing only to prescribing and dispensing, therefore there are expected changes to workflow in community pharmacies that choose to provide the service. This service would take community pharmacists away from their dispensing and other duties; therefore, payment for pharmacist services will be necessary for employers to allow and encourage participation. Additionally, barriers related to pharmacy space and logistics need to be addressed by each pharmacy to increase the likelihood of participation. Similar findings were noted in survey studies of pharmacist intentions to participate in the new authority in California and Oregon after the state laws were passed and prior to protocol development [10,11].

Previous studies explored attitudes of women, student pharmacists, pharmacists and other providers regarding direct pharmacy access to contraception, however this study adds new information to existing literature since it explored pharmacist attitudes immediately following the first state law that set the precedent for pharmacist prescribing of contraception and possible barriers to implementation in pharmacies [3,8,9,10,11,16,17]. These interviews were completed in 2014 and given this recent expansion in the scope of practice and education efforts surrounding it, particularly since the protocol became available in 2016, it is expected that there is more knowledge, more perceived need, and more acceptance of this service as part of the pharmacist’s role. 

Following implementation of statewide pharmacist prescribing protocols, further studies would be beneficial to re-evaluate implementation barriers and facilitators, as well as understanding the utilization and impact on both patients and pharmacies.

## 5. Conclusions

Our study demonstrates community pharmacists foresee challenges with implementing pharmacist prescribing of hormonal contraception in pharmacies. Barriers will need to be addressed and requisite training disseminated widely to facilitate successful implementation and thus improve access on a broad scale. Further research following protocol implementation is needed to understand service implementation, as well as patient utilization and satisfaction.

## Figures and Tables

**Table 1:** Demographic characteristics of pharmacists interviewed (N = 30).

Characteristic	n (%)
**Gender**	
Male	15 (50%)
Female	15 (50%)
**Age**	
<30	7 (23.3%)
31–40	11 (36.7%)
41–50	2 (6.7%)
51–60	2 (6.7%)
>60	8 (26.7%)
**Years in practice**	
<5	11 (36.7%)
6–10	5 (16.7%)
11–15	2 (6.7%)
16–20	1 (3.3%)
>20	11 (36.7%)
**Role in pharmacy**	
Staff	12 (40%)
Manager	16 (53.3%)
Owner	0 (0%)
Other (floater)	2 (6.7%)
**Completed a pharmacy residency**	
Yes	3 (10%)
No	27 (90%)
**Attended a California pharmacy school**	
Yes	17 (57%)
No	13 (43%)
**Trained to prescribe emergency contraception**	
Yes	13 (43%)
No	17 (57%)
**Pharmacy location**	
Urban	15 (50%)
Suburban	10 (33.3%)
Rural	5 (16.7%)
**Type of community pharmacy**	
Chain	20 (66.7%)
Independent	8 (26.7%)
Other (community clinic)	2 (6.7%)
**Proportion of hormonal contraception among total prescriptions**	
<1%	4 (13.3%)
1–5%	15 (50%)
6–10%	8 (26.7%)
11–25%	2 (6.7%)
>25%	1 (3.3%)
Amount of emergency contraception prescribed under protocol * per week	
1 or less	11 (36.7%)
2 or more	2 (6.7%)
**Amount of emergency contraception sold over-the-counter per week**	
0–10	26 (86.7%)
11–20	2 (6.7%)
21–30	0 (0%)
31–40	0 (0%)
41–50	1 (3.3%)
>50	1 (3.3%)

* Protocol is referring to the California Board of Pharmacy Emergency Contraception Protocol (Section 1746; under the authority of California Business and Professions Code 4052.3) allowing a pharmacist to prescribe (also known as furnish) oral emergency contraception to a patient following training.

## Appendix A

**Interview Guide**

**Pharmacist Prescribing of Emergency Contraception (EC)**

1. Can you tell us about your decision to become or not become emergency contraception or EC-certified, allowing you to prescribe EC under the California Board of Pharmacy protocol?

**Sample Patient Scenarios**

2. What are some challenges you currently see with dispensing hormonal contraception?
3. First example: You see the same patient coming in for emergency contraception on multiple occasions.
4. Second example: A patient is late refilling her birth control and has no refills left.
5. Third example: A patient is filling a prescription for a new medication, topiramate, and you notice she has birth control pills on her profile.
6. Fourth example: You have an obese woman asking for emergency contraception.
7. Fifth example: A 17 year old is interested in starting contraception.

**Long-Acting Reversible Contraception**

8. Are you familiar with long-acting reversible contraceptive methods? Can you tell me which methods fall into this category?
9. Do you talk to patients about these methods?

**Pharmacist Prescribing of Hormonal Contraception**

10. Are you familiar with SB493 and the new authority related to contraception?
11. Do you feel this is safe?
12. Which patients do you think would use this service or benefit from it the most?
13. Are you interested in participation or planning on participating in this new authority and providing hormonal contraception directly to patients?
14. What motivates your interest or lack of interest to participate?
15. What challenges to you see in providing this service?
16. Do you feel you are equipped and comfortable measuring a patient’s blood pressure in your current pharmacy workplace?
17. Would you feel comfortable using a screening tool to determine a woman’s candidacy for the various contraceptive methods?
18. For which aspects do you feel you need further support or education on in order to implement this new service in your pharmacy workplace?
19. Can services and patient counseling be provided confidentially at your workplace?
20. How do you feel about the reimbursement issues for this pharmacist service?
